# Deep Learning-Based Diagnosis Algorithm for Alzheimer’s Disease

**DOI:** 10.3390/jimaging10120333

**Published:** 2024-12-23

**Authors:** Zhenhao Jin, Junjie Gong, Minghui Deng, Piaoyi Zheng, Guiping Li

**Affiliations:** College of Electrical and Information, Northeast Agricultural University, 600 Changjiang Road, Harbin 150038, China; jzh1861@163.com (Z.J.); gongjunjiee@126.com (J.G.); zpysyx@163.com (P.Z.); liguiping157@163.com (G.L.)

**Keywords:** Alzheimer’s disease, deep learning, automatic auxiliary diagnosis, MRI, attention mechanism

## Abstract

Alzheimer’s disease (AD), a degenerative condition affecting the central nervous system, has witnessed a notable rise in prevalence along with the increasing aging population. In recent years, the integration of cutting-edge medical imaging technologies with forefront theories in artificial intelligence has dramatically enhanced the efficiency of identifying and diagnosing brain diseases such as AD. This paper presents an innovative two-stage automatic auxiliary diagnosis algorithm for AD, based on an improved 3D DenseNet segmentation model and an improved MobileNetV3 classification model applied to brain MR images. In the segmentation network, the backbone network was simplified, the activation function and loss function were replaced, and the 3D GAM attention mechanism was introduced. In the classification network, firstly, the CA attention mechanism was added to enhance the model’s ability to capture positional information of disease features; secondly, dilated convolutions were introduced to extract richer features from the input feature maps; and finally, the fully connected layer of MobileNetV3 was modified and the idea of transfer learning was adopted to improve the model’s feature extraction capability. The results of the study showed that the proposed approach achieved classification accuracies of 97.85% for AD/NC, 95.31% for MCI/NC, 93.96% for AD/MCI, and 92.63% for AD/MCI/NC, respectively, which were 3.1, 2.8, 2.6, and 2.8 percentage points higher than before the improvement. Comparative and ablation experiments have validated the proposed classification performance of this method, demonstrating its capability to facilitate an accurate and efficient automated auxiliary diagnosis of AD, offering a deep learning-based solution for it.

## 1. Introduction

Alzheimer’s disease (AD) poses a significant threat to human health. It is a neurodegenerative disorder that begins insidiously in late adulthood. Patients with AD experience a gradual decline in memory, loss of cognitive abilities, and reduced language expression and comprehension, leading to a loss of independence and the need for specialized care, imposing a heavy burden on both patients and their families [1,2,3]. Projections estimate that by 2050, the global AD patient population will reach 123 million, accompanied by a staggering estimated treatment cost of USD 18,871.8 billion, exerting a profound impact on nations and societies [4].

Numerous scholars and experts have explored the field of automated Alzheimer’s disease (AD) diagnosis, conducting extensive research. They traditionally relied on manual methods to identify features from data, which were then inputted into conventional machine learning algorithms for classification. These manually extracted features were based on specialized knowledge and experience, targeting specific brain regions believed to harbor discriminative information. For instance, Achterberg et al. [5] and Plocharski et al. [6] selected hippocampal shape or sulcal length as features and applied machine learning classifiers to categorize AD cases. Stonnington et al. [7], for example, investigated the relationship between MR images of AD patients and clinical scores using associative vector regression, establishing a model to predict clinical scores from a single MRI scan. However, due to the complexity of AD, traditional feature extraction methodologies have proven insufficient for achieving high-precision diagnoses. To further enhance the accuracy of AD classification, researchers have combined multiple image features to improve classification performance. Pan et al. [8], for instance, considered regional properties and connectivity, devising a method using single-modality fluorodeoxyglucose positron emission tomography (FDG-PET) data but with multi-level features. Zheng et al. [9] conducted principal component analysis on brain connectivity regions from multimodal images, pinpointing key discriminative locations in the brain. Ramírez et al. [10] improved random forest performance in NC and MCI classification by implementing a hierarchical classifier approach, where additional features were selected for reclassification of poorly classified instances at the first level. Altaf et al. [11] trained AD classifiers by use of an SVM, random forest, and K-nearest neighbors (KNN) algorithms, assigning weights to each classifier and aggregating their outputs for a final classification decision.

Deep learning has exhibited remarkable capabilities in the automatic auxiliary diagnosis of Alzheimer’s disease (AD). Compared to conventional diagnostic methods, deep learning algorithms demonstrate significantly greater efficacy in enhancing the precision and efficiency of Alzheimer’s disease (AD) diagnosis. Based on the characteristics of Alzheimer’s disease, this paper proposes a three-classification network of MCI (mild cognitive impairment: this group has mild cognitive impairment, where individuals show minor memory or cognitive issues that have not yet progressed to Alzheimer’s disease. MCI may or may not advance to Alzheimer’s over time)/NC (normal control: this represents the normal control group, consisting of individuals with normal cognitive function, used as a baseline for comparison against those with cognitive impairments)/AD (Alzheimer’s disease: this category includes individuals diagnosed with Alzheimer’s disease, representing the most severe stage of cognitive decline). In the segmentation network, the backbone network was simplified, the activation function and loss function were replaced, and the 3D GAM attention mechanism was introduced. In the classification network, firstly, the CA attention mechanism was added to enhance the model’s ability to capture the positional information of disease features; secondly, dilated convolutions were introduced to extract richer features from the input feature maps; and finally, the fully connected layer of MobileNetV3 was modified and the idea of transfer learning was adopted to improve the model’s feature extraction capability. In actual testing, the model showed excellent performance.

The structure of this paper is organized as follows: the first section introduces the background, significance, and innovation of this research. The second section reviews related work, providing an overview of relevant studies. The third section, Materials and Methods, describes in detail the dataset used and the proposed methodology. The fourth section presents the experiments and results, outlining the experimental setup, procedures, and analysis of results. The fifth section provides a discussion, offering an in-depth analysis of the findings, limitations of this study, and potential future directions. The sixth section concludes the paper by summarizing the main contributions and the significance of this work.

## 2. Related Work

Researchers have integrated deep learning with traditional machine learning algorithms, utilizing deep learning for feature extraction from brain images and machine learning for subsequent feature classification. Sharma et al. [12] proposed the FDN-ADNet model for early AD diagnosis, which extracts features using a deep residual network (ResNet-101) and classifies AD using a fuzzy least squares twin support vector machine (FLS-TWSVM) based on a blurred hyperplane. Zhang et al. [13] proposed a 3D end-to-end generative adversarial network (BPGAN) model for multimodal AD diagnosis, thereby enhancing the adaptability of the model to diverse medical scenarios.

As deep learning neural network models demonstrate superior performance in various classification tasks, researchers have applied these models to Alzheimer’s disease (AD) feature classification, thereby enhancing the efficacy of AD classification algorithms. Hazarika et al. [14], addressing the slow computation speed of DenseNet models due to numerous convolution operations, proposed replacing all convolutional layers in DenseNet-121 with deep convolutional layers. Neelum et al. [15] used the Inception-v3 model to identify brain tumors with an accuracy of 94.34%. Neelum et al. [16] used a feature concatenation method based on a pre-trained model in brain tumor detection, with detection accuracy rates of 99.34% and 99.51% for the test samples, respectively. Najmul et al. [17] used a multi-stage deep neural network structure based on a residual function to identify Alzheimer’s disease and achieved high recognition accuracy. Liu et al. [18,19] utilized depthwise separable convolutional networks, significantly reducing the number of parameters and the computational costs of neural networks, making them suitable for edge devices like mobile platforms and facilitating portable AD automatic auxiliary diagnosis solutions with lightweight deep learning models.

To further boost the overall performance of models, researchers have combined neural network models with other optimization strategies. Kumar et al. [20] proposed selecting the most informative MRI slices using entropy image slicing during training, along with a VGG-16 network fine-tuned via transfer learning for AD classification. As demonstrated by Liang et al. [21], who leveraged LSTM in a multi-task learning framework to predict AD progression based on correlations across different time points. Du et al. [22] incorporated attention mechanisms into convolutional neural networks to capture key regions in AD classification tasks, though this multi-task-, multi-data-type learning increases model complexity and parameter counts. Hu et al. [23] proposed an attention-based 3D RepVGG network, combining RepVGG architecture with 3D convolutions and a 3D SE attention mechanism. Wang et al. [24] employed Faster R-CNN, utilizing dense feature pyramid networks for multi-scale feature extraction and CURE clustering for anchor box optimization, achieving a 96.9% classification accuracy on AD and NC datasets, as shown by Pan et al. [25], who augmented PET data using GANs and exploited the synergies between MRI and PET data for AD diagnosis assistance. Alsubaie et al. [26] applied convolutional neural networks (CNNs), recurrent neural networks (RNNs), and generative models to detect Alzheimer’s disease, exploring future directions for deep learning methods in this field. Sait et al. [27] enhanced Alzheimer’s disease identification by modifying the existing vision transformer (ViT) architecture to improve the feature extraction process, thereby increasing model accuracy for AD detection. Ganesan et al. [28] used a capsule network to detect Alzheimer’s disease and achieved high accuracy. Ali et al. [29] used a canonical correlation approach (CCA) to merge the extracted image information from deep networks and then used binary enhanced WOA for optimal feature selection, achieving high accuracy.

## 3. Materials and Methods

### 3.1. Dataset and Preprocessing

**Figure 1:**
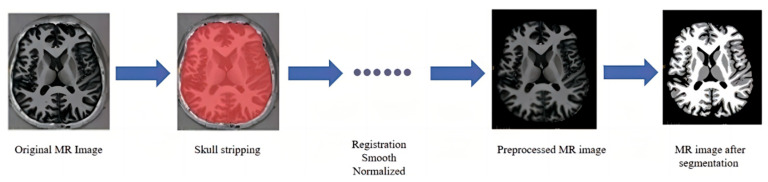
Brain MR image preprocessing.

The brain MRI image segmentation dataset is derived from the brain MR Image database in collaboration with the IDEA Laboratory at the University of North Carolina at Chapel Hill, USA. Data were collected from a total of ten subjects and corresponding T1-weighted MRI images were also obtained from a 3T MRI scanner. Considering the different backgrounds of acquired brain MR image data collection, preprocessing operations must be performed on all the acquired data, such as removing noise, removing the cerebellum and brainstem, and performing registration. The preprocessing process is illustrated in Figure 1. In order to ensure that the segmentation model can be fully trained and learned, the collected data are expanded. Considering that these are brain MRI data, the data are extended by basic horizontal and vertical inversion and rotation methods.

In this paper, the collected data in the ADNI database were enhanced based on the DAGAN model, then the brain tissue of the MR image was segmented based on a 3D DenseNet segmentation model, and finally, the dataset for the AD classification experiment was constructed. Three-dimensional MRI data must be extracted and sliced before training. A total of 18250 coronal slices were obtained from the 605 3D datasets obtained for model training and testing. The data of training set, validation set, and test set are guaranteed to be independent of each other. Among them, the ratio of data volume of the training set to the data volume of the validation set to the data volume of test set is 64%: 16%: 20%, and the specific number of allocated data points is shown in Table 1.

### 3.2. Convolutional Neural Network Models

**Figure 2:**
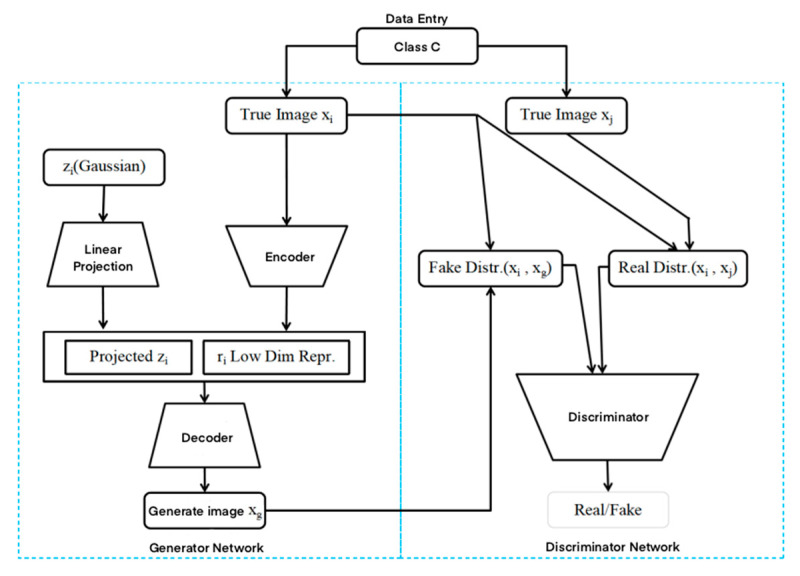
DAGAN structure.

The generator of DAGAN is a combination of UNet and ResNet networks, which can be called UResNet. The UResNet generator has a total of 8 blocks, each with 4 convolutional layers, followed by a downsampling layer and an upsampling layer. The downsampling layer is a convolution with a stride of 2, followed by a Leaky ReLu activation function, batch normalization, and a Dropout layer. The upsampling layer is a replicator with a stride of 1/2, followed by a convolution, a Leaky ReLu activation function, batch normalization, and Dropout. In addition, each block of the UResNet generator has skip connections. Like the standard ResNet, the strided 1 × 1 convolution also transfers information between blocks, bypassing the nonlinearity between blocks to help the gradient flow. Finally, skip connections are introduced between filters of equivalent size at each end of the network (same as UNet). DAGAN uses DenseNet as the discriminator and uses layer normalization, LN. DenseNet consists of 4 dense blocks and 4 transition layers, with a growth rate of k = 64. There are 4 convolutional layers in each dense block, and a Dropout layer is used in the last convolutional layer of each dense block to improve the quality of the samples. The DAGAN structure is shown in Figure 2.

**Figure 3:**
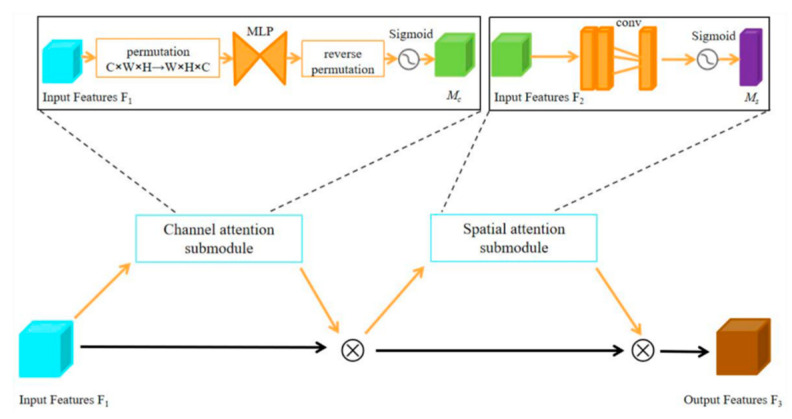
The workflow flowchart of 3D GAM.

In this study, the method of first segmentation and then classification is used to realize the automatic auxiliary diagnosis of AD. In the segmentation stage, by a simplified backbone of dense connection redundancy in the 3D DenseNet model, the focal loss function is used instead of the cross-entropy function to strengthen the model’s learning of positive samples, and the Leaky ReLU function is used instead of the ReLU function to avoid the problem of neuron death in training. The 3D GAM attention mechanism was added to the network output to improve the model’s ability to distinguish WM, GM, and CSF. The GAM adopts a channel-first mechanism, then a spatial attention mechanism sequence similar to that in CBAM but with a redesigned channel and spatial submodules. In the channel attention submodule, a three-dimensional arrangement is used to preserve 3D information, followed by a two-layer multi-layer perceptron (MLP) to amplify the cross-dimensional channel–spatial dependencies. The spatial attention submodule focuses more on spatial information, utilizing two convolutional layers for spatial information fusion. Due to the negative impact of pooling on spatial information, this submodule eliminates the pooling layers. Additionally, the module employs group convolutions with channel shuffling to prevent an increase in parameters. The workflow of the 3D GAM attention mechanism is shown in Figure 3. Given the input feature map *F*_1_, the intermediate feature map *F*_2_, and the output feature map *F*_3_, they are defined as follows:

(1) $$F_{2}=M_{c}(F_{1})⊗F_{1}$$

(2) $$F_{3}=M_{s}(F_{2})⊗F_{2}$$

**Figure 4:**
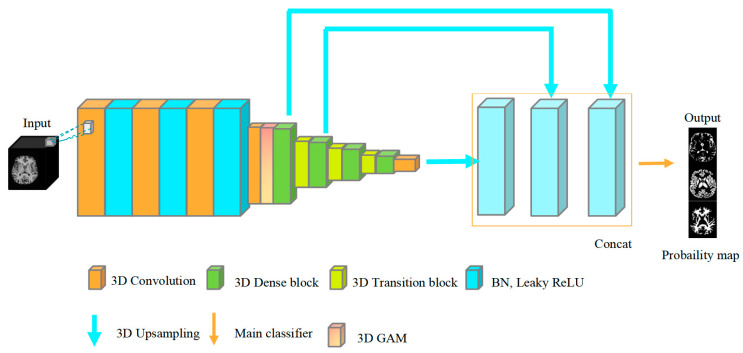
Improved 3D DenseNet model structure.

The 3D DenseNet model provides a dense connection between layers designed to improve the flow of information in the network. Figure 4 shows the structure of the improved 3D DenseNet model. Among them, the improved model first uses three 3D convolutions to extract features, and the following network mainly includes two paths, namely downsampling and upsampling. Among them, the downsampling path refers to the cooperative work between four dense blocks after three convolution operations. Each 3D dense block includes a process such as BN → Leaky ReLU →1 × 1 × 1 convolution → BN → Leaky ReLU →3 × 3 × 3 convolution, which mainly serves to reduce the resolution of feature maps, increasing the receptive field and improving computational performance. After each 3 × 3 × 3 convolution, the Dropout layer is used to solve the overfitting problem. Then, there is the 3D transition block between adjacent dense blocks, in which the two-dimensional 1 × 1 convolution correspondingly becomes a three-dimensional 1 × 1 × 1 convolution and the subsequent pooling layer is replaced by the convolution layer with the stride of 2 so as to solve the problem of easy loss of spatial information after the use of a pooling layer.

The upsampling path, namely the 3D upsampling operation, is used to restore the resolution of the input feature map. The 3D upsampling operation is performed after the dense block is executed, and the output feature map obtained after the upsampling is spliced through concat. Because the shallower layers contain local features and the deeper layers contain global features, the combination of feature maps from different levels can capture multi-scale context information for more accurate predictions. A classifier consisting of 1 × 1 × 1 convolution classifies the concatenated feature maps into three classes of the brain (CSF, WM, GM). Finally, the probability map of brain tissue was obtained using Softmax classification.

In the classification stage, aiming at the problems of time-consuming feature extraction and low classification accuracy in traditional AD diagnosis methods, this paper proposes an improved MobileNetV3 classification model to realize AD automatic classification. First, the AD classification dataset is constructed, and the data are enhanced based on DAGAN. Then, the segmented images are obtained based on the improved 3D DenseNet model, and the segmented images are realized by the improved MobileNetV3 model for AD classification.

The MobileNetV3 [30] network model is another deep learning model in the MobileNets series proposed by Google in 2019. The core module of the MobileNetV3 model is the Bneck module. This module mainly realizes the deep separable convolution of V1 + inverse residual structure with a linear bottleneck of V2 + SE channel attention mechanism, and it uses activation functions such as Hard-Swish to optimize the network structure so as to improve the accuracy of the model. The Hard-Swish activation function is improved from the Swish function, which has the characteristics of no upper bound and lower bound and being smooth and non-monotonic. The Swish function [31] is better than the ReLU [32] function on deep models, and its expression is as follows:

(3) f(x)=x ∗ sigmoid(βx)

The Hard-Swish function is mainly an improvement to the Sigmoid operation of the Swish function, which is not friendly to hardware. Its expression is as follows:

(4) $$\mathrm{Hardswish}(x)=\left\{\begin{array}{ll} 0 & if\;x\leq -3\\ x & if\;x\geq +3\\ x\cdot (x+3)/6 & \mathrm{otherwise} \end{array}\right.$$

In order to comprehensively improve the performance of the AD classification experiment, this paper makes improvements based on the MobileNetV3 network model, which mainly includes the following aspects:

First of all, the SE attention mechanism is used in the MobileNetV3 model, and SE attention focuses on the information between channels but ignores the location information. Therefore, in order to improve the accuracy of the model, the specific operation is to replace the SE module in the Bneck structure of the MobileNetV3 model with the CA attention module [33]. It can help MobileNetV3 model quickly and accurately locate the most effective feature information in AD brain MR images.

Secondly, cavity convolution is introduced in the first and last Bneck modules of the MobileNetV3 network. Dilated convolution (DC) [34] is a way to expand the receptive field of conventional convolution. From the point of view of feature extraction, the region of convolution operation is expanded by adding cavities, so that richer features can be extracted from the input feature maps.

In order to improve the efficiency of the model and further improve the ability of the model to extract features, the idea of transfer learning was adopted, and MobileNetV3 was pre-trained on the ImageNet dataset. The pre-trained model effectively learns a range of low- and mid-level visual features, such as edges, textures, and shapes, which provide a solid foundation for Alzheimer’s disease classification. Next, the MobileNetV3 model, trained on the ImageNet dataset, is applied to this classification task. In this process, rather than initializing the model’s weights randomly, the weights from the ImageNet pre-training are retained. This transfer allows the model to build upon its prior knowledge, thereby enhancing its feature extraction capability for this specific classification problem. Transfer learning applies to deep neural networks that use a small amount of training data, applying a model trained on one task to a related task by using a “fine-tuning” approach to adjust the pre-trained model on a larger training dataset to reduce training time.

The loss function is used to measure the deviation of the predicted value from the real value of the model. The better the loss function is, the better the performance of the model is generally. Through this degree of deviation, the predicted value is close to the real value so as to achieve the purpose of learning. In this paper, the cross-entropy loss function is used to solve the loss.

**Figure 5:**
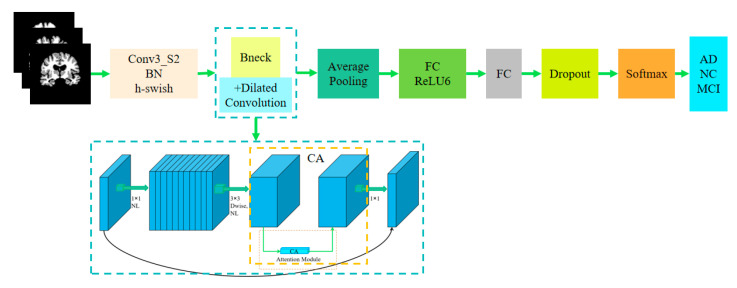
Improved MobileNetV3 structure.

In model training, fitting problems are often encountered. In order to prevent overfitting, Dropout is often introduced into the CNN model. The researchers found that there were also differences between neurons in the neural network, and some neurons had higher predictive power, resulting in excessive dependence on one neuron. To avoid these effects, the weights need to be adjusted by regularization methods to prevent overfitting. Dropout [35] is one such approach, which temporarily and randomly ignores a subset of neurons by changing their values to 0, enabling immediate corrections in network feature extraction, thereby enhancing the model’s generalization performance. Based on this, in order to enhance the generalization of the model, the Dropout layer is added after the last fully connected layer of the MobileNetV3 network. Finally, the extracted features are used in Softmax classifier training to obtain AD classification results. Based on the above improvement methods, the improved MobileNetV3 model structure can be obtained, as shown in Figure 5.

**Figure 6:**
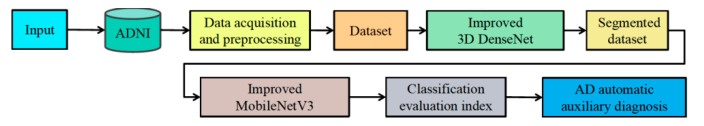
AD automatic auxiliary diagnosis algorithm based on improved MobileNetV3.

The improved MobileNetV3 model is applied to the automatic classification experiment of AD, and the slice data from the 3D DenseNet segmentation model is recognized so as to help realize the automatic diagnosis of AD. Based on this, an AD automatic auxiliary diagnosis algorithm framework based on the improved 3D DenseNet segmentation model and MobileNetV3 classification model is constructed, as shown in Figure 6.

The fully connected layer of the MobileNetV3 model is improved; after the average pooling layer in the original MobileNetV3 model, entering the fully connected layer requires two 1 × 1 convolution layers, and the Hard-Swish activation function is used to output the result at last. However, the derivation of the Hard-Swish activation function is complicated, which increases the calculation cost, while the ReLU6 activation function has the advantage of a simple and fast calculation. Based on this, this paper proposes to replace the Hard-Swish activation function with the ReLU6 activation function. The ReLU6 function is derived from the ReLU function, which limits the maximum output value of the ordinary ReLU function to 6 in order to reduce the loss of quantization accuracy. The expressions of the two are shown in Formulas (5) and (6).

(5) ReLU=max(0,x)

(6) ReLU6=min(6,max(0,x))

### 3.3. Evaluating Indicators

To evaluate the effect of segmentation, this study used the dice ratio to quantitatively measure the accuracy of segmentation. The equation is shown in (7) [36]. Specifically, let *A* and *B* denote the binary images generated by the manual and predictive segmentation of a tissue category on the pixels of a specific object, respectively. |*A*| represents the total number of pixels of each label in the golden standard segmentation image *A* and |*A* ∩ *B*| represents the total number of pixels in the intersecting part of the manual segmentation and prediction. It can be observed that the dice ratio is the volume overlap rate, and the value range is [0, 1]. The closer the value is to 1, the closer the segmentation result of the model is to the artificial segmentation result and the better the model effect, and vice versa.

(7) $$Dice=\frac{2|A\cap B|}{\left|A|+|B\right|}$$

In the classification experiment of Alzheimer’s disease, in order to quantify the performance of network models, the following common evaluation indicators were used to evaluate the performance of the Alzheimer’s disease classification algorithm: accuracy (*Acc*), sensitivity (*Sens*), specificity (*Spec*), F1 score (*F*1), and AUC values. A confusion matrix could be obtained based on the relationship between the predicted and true values used to show the classification results.

The accuracy indicates how many disease predictions are correct, as shown in Formula (8).

(8) $$Acc=\frac{TP+TN}{TP+FP+FN+TN}$$

Sensitivity represents the proportion of correctly identified diseased samples in the total diseased samples. The sensitivity is equal to the recall rate (*Recall*), and the formula is shown in Formula (9).

(9) $$Sens=\frac{TP}{TP+FN}$$

Specificity represents the proportion of the correctly identified healthy samples in the total number of healthy samples (10).

(10) $$Spec=\frac{TN}{TN+FP}$$

Precision is the evaluation of the correct rate of a certain category predicted by the classifier (11).

(11) $$Precision=\frac{TP}{TP+FP}$$

The *F*1 score, taking into account both the accuracy rate and recall rate, is a comprehensive evaluation index. The formula is shown in (12).

(12) $$F1=\frac{2Precision\times Recall}{Precision+Recall}$$

AUC value: The AUC (area under the ROC curve) is the probability that the probability of predicting the true value of the disease data is greater than the probability of predicting the true value of the health data. The value of the AUC is the area under the receiver operating characteristic curve (ROC). The ROC curve draws the relationship between specificity and sensitivity and is not affected by a threshold, which can objectively measure the performance and effect of the model. The closer the value of the AUC is to 1, the better the model performance is.

## 4. Experiments and Results

### 4.1. Experimental Environment and Platform

This research was conducted using the PyTorch deep learning framework, which features a dynamic computational graph that is highly flexible and fast. It also has a GPU tensor that uses GPU acceleration to process big data in a short time, thus speeding up training. This section was completed in Ubuntu16.04 and the programming language was Python3.7. The model was trained from scratch, with 200 training epochs. The first 50 epochs were frozen training, and only the network backbone was fine-tuned. Mosaic and mixup were used to enhance the training data. Adam was used as the optimizer. The initial learning rate was set to 0.01, the momentum was 0.937, the weight decay was 5 × 10^−4^, and the cosine annealing algorithm was used to dynamically optimize the learning rate. With the Adam optimizer, the accuracy of the improved model reached a high level after multiple training and testing sessions. All models were trained on an NVIDIA GTX 4090 GPU. The device has 64 GB of video memory, and the batch size was set to 50 based on the size of the memory. Bayesian optimization was used to optimize hyperparameters to achieve near optimal results with fewer experiments.

### 4.2. Experiment Results and Analysis of Segmentation Model

Sub in Table 2 represents the subject of experimental training. As can be seen from the table, the segmentation effect of the segmentation model for white matter is generally higher than that for gray matter and cerebrospinal fluid, and the segmentation effect of cerebrospinal fluid is the worst. The main reason is that the three types of brain tissue have different degrees of difference, in which the white matter tissue has the most obvious difference, followed by the gray matter, and the cerebrospinal fluid is the closest to the background color, so the model has the least ideal segmentation effect for cerebrospinal fluid.

Table 3 describes the average Dice coefficient of brain image tissue segmentation obtained by the segmentation of brain MR image segmentation data based on different segmentation methods. It can be seen from the table that the improved 3D DenseNet model can achieve high-level brain MR image segmentation tasks with average Dice coefficients of 0.9305 (GM), 0.9778 (WM), and 0.8951 (CSF), respectively, and an overall average segmentation accuracy of 0.9345. In contrast, the overall average Dice coefficients of FSL, MIPAV, and SPM are 0.7921, 0.7917, and 0.7607, respectively, and the accuracy of the proposed segmentation model is about 1417 percentage points higher than that of these. Therefore, the improved 3D DenseNet segmentation algorithm model proposed in this paper is superior to the traditional automatic segmentation tools in terms of accuracy. Of the three kinds of tissue segmentation effects, the segmentation effect of the white matter is the best, the gray matter is the second best, and the cerebrospinal fluid is the worst.

By longitudinal observation of the experimental results in Table 3, it can be found that the average segmentation results of the improved 3D DenseNet segmentation model on the three types of brain tissues of the sample data and the whole brain tissues are not only higher than the traditional medical image segmentation software but also better than the random forest segmentation model and the classic deep learning segmentation model U-Net. At the same time, compared with the original segmentation model before the improvement, the overall segmentation accuracy is improved by about 2.2 percentage points, indicating that the improved model can achieve a higher accuracy of brain tissue segmentation. Compared with the machine learning segmentation algorithm model based on random forest, the deep learning neural network model has improved the overall segmentation of brain MR images by about 5.2 percentage points, which has significant improvements and advantages. The comparison of experimental results shows that the deep learning neural network model can effectively distinguish three kinds of brain tissue and improve the segmentation accuracy of brain MR images. Thus, the automatic segmentation of brain MR images can be realized efficiently.

**Figure 7:**
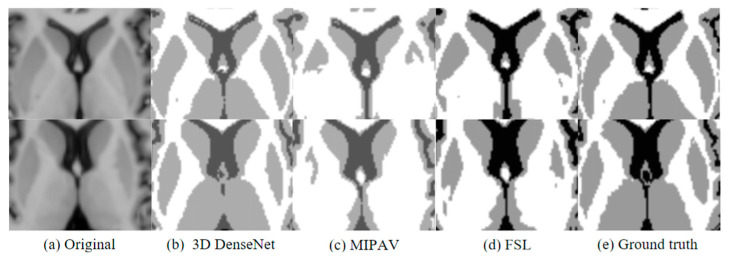
Local magnification and comparison of segmentation slices.

Figure 7 shows four sequential slices of the brain MR images of the subject’s sample data after segmentation by different segmentation methods. In the figure, the first column is the four slice graphs of the original T1-weighted image, the fifth column is the truth value image manually segmented by experts, and the second, third, and fourth columns are the results of the sample segmentation by applying the improved 3D DenseNet segmentation algorithm, MIPAV, and FSL, respectively.

**Figure 8:**
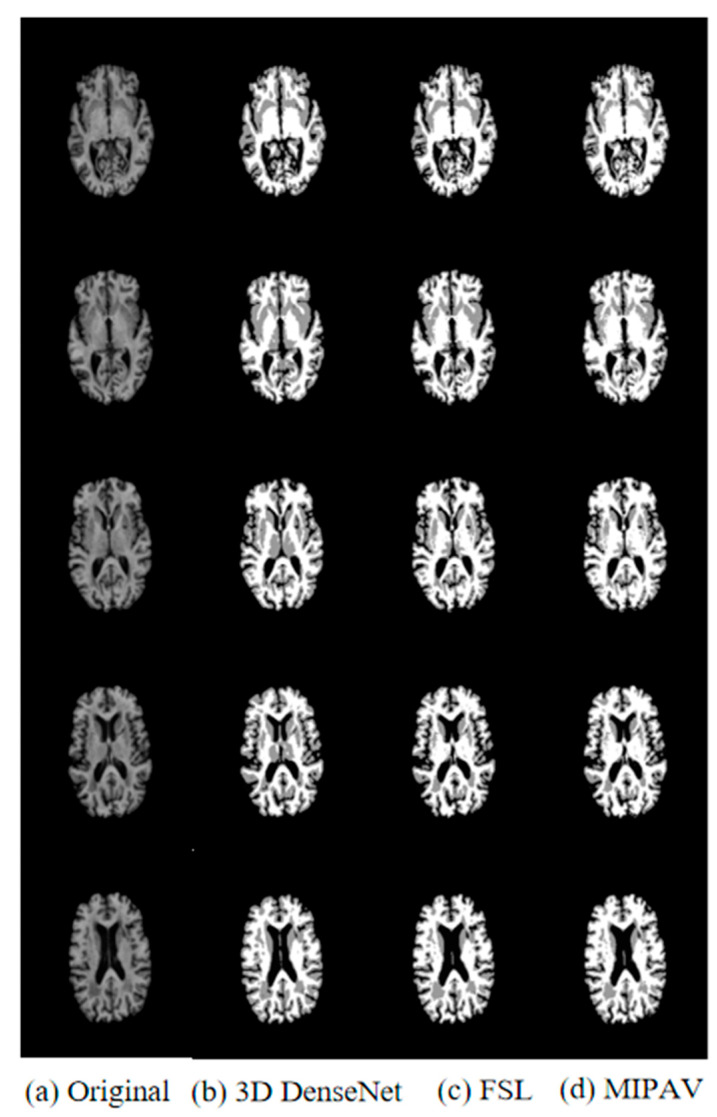
Comparison of segmentation results on ADNI dataset with different methods.

Figure 8 compares the results of the segmentation of data obtained from ADNI datasets based on different methods. The first column shows five sequences of the original T1-weighted image, and the second column shows five sequences of the proposed improved 3D DenseNet segmentation model. The third and fourth columns show the results of segmentation using FSL and MIPAV traditional automatic segmentation software, respectively. As can be seen from the figure, the segmentation method proposed in this paper is relatively more complete, clearer, and more accurate in the segmentation of images in ADNI datasets, and the segmentation effect is better than that of using medical software tools. The application of the 3D DenseNet segmentation model to AD diagnosis can improve the performance of the algorithm more effectively. Therefore, the improved 3D DenseNet segmentation model is used to segment the obtained classification data before AD classification.

### 4.3. Binary Classification Experiments

**Figure 9:**
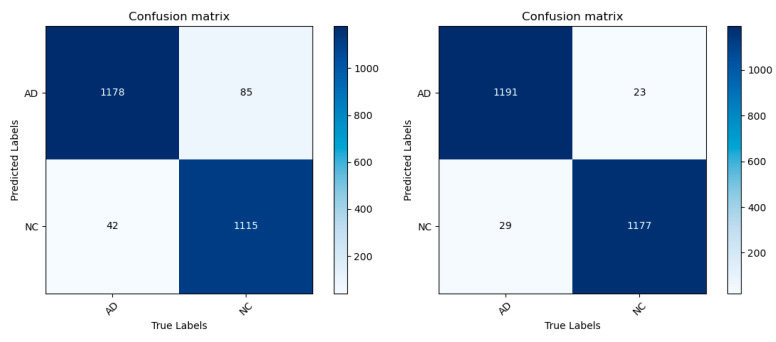
AD/NC confusion matrices before and after model improvement.

**Figure 10:**
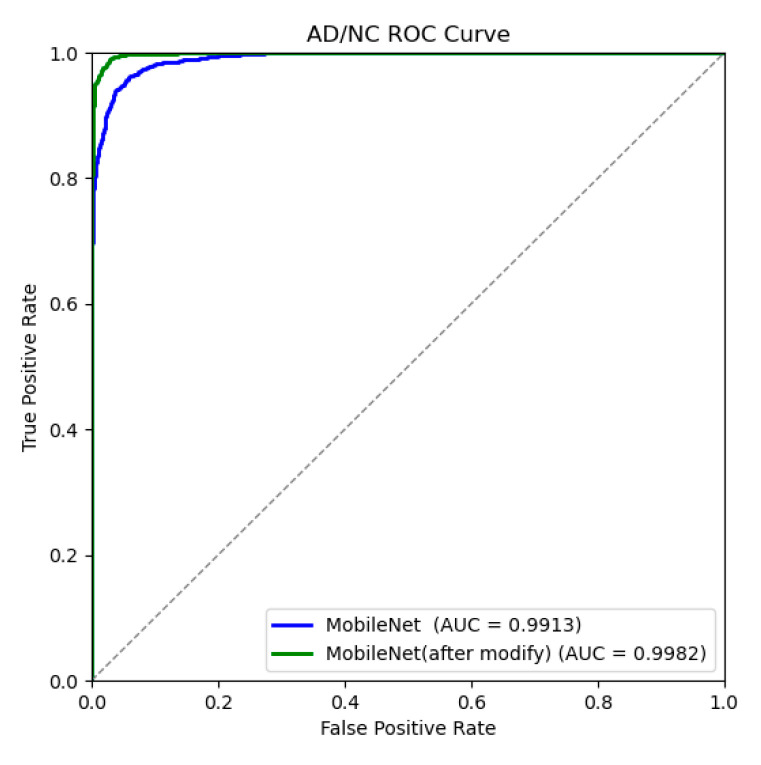
AD/NC ROC curve before and after model improvement.

In order to intuitively analyze the performance of the MobileNetV3 model, the corresponding confusion matrix and ROC curve were drawn, as shown in Figure 9 and Figure 10, clearly presenting the relevant experimental results of the AD/NC binary classification. In the confusion matrix, the horizontal represents the true value and the vertical represents the predicted value. Based on this confusion matrix, four indicators can be derived, which are accuracy, precision, recall, and the F1 score. The area of the ROC curve is called the AUC, which measures the proportion of the model’s “gain” and “cost” in identifying positive classes at all probability thresholds. Therefore, the larger the AUC value, the better, with a range of values [0, 1]. Table 4 calculates the specific values of each classification evaluation index corresponding to the improved MobileNetV3 network model. As can be seen from the table, the improved MobileNetV3 model shows a very high evaluation index performance compared with that before the improvement. The accuracy rate of the improved model reaches 97.85%, which is 3.1 percentage points higher than that of the improved model. In addition, the specificity is 98.08%, the accuracy is 98.11%, and the F1 score is 97.86%, which are significantly improved from the indicators of the improved model, indicating that the improved model has enhanced the recognition ability of AD and NC. There is a slight increase in both the parameter count and computational complexity, but this is justified by the corresponding improvement in accuracy. The AUC value can reach 99.82%, which is close to 1, indicating that the improved MobileNetV3 model has a very good classification performance in AD/NC classification tasks. The improved model not only shows a very high accuracy of AD classification but also can well identify and distinguish the feature differences between AD and NC, and the number of missed and false checks in the model is very small, so it can effectively realize the accurate classification of AD and NC.

MCI is a transitional state between AD and NC. In order to achieve a better automatic diagnosis of MCI, it is also very important to accurately distinguish MCI patients from NC patients and AD patients from MCI patients, which can not only greatly improve the possibility of preventing MCI patients from converting into AD patients but also provide patients with the correct treatment plan in time. The efficiency of AD diagnosis can be improved. Therefore, binary classification experiments of brain MR images with MCI and NC and AD and MCI are needed.

**Figure 11:**
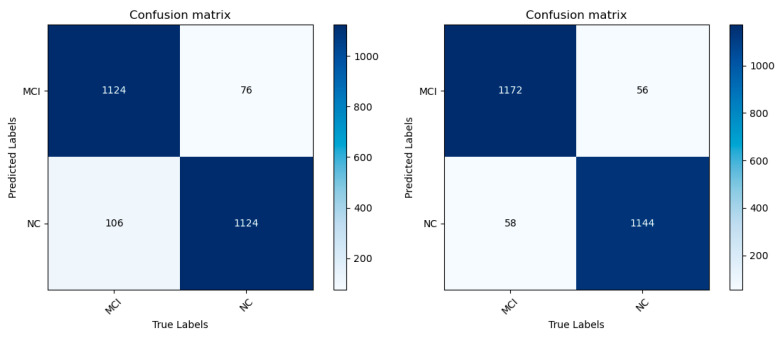
MCI/NC confusion matrices before and after model improvement.

**Figure 12:**
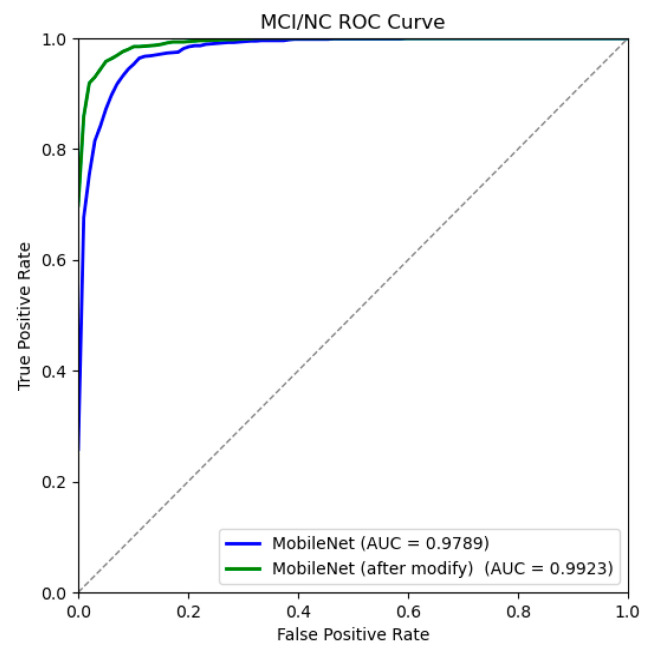
MCI/NC ROC curve before and after model improvement.

According to the confusion matrix and ROC curve of MCI/NC binary classification experiment (Figure 11 and Figure 12), Table 5 is drawn to summarize the numerical results of each classification evaluation index. As can be seen from the table, all indexes of the MCI/NC classification experiment based on the improved MobileNetV3 model show high classification performance. Among them, the accuracy of the improved model reached 95.31%, 2.8 percentage points higher than that before the improvement, the sensitivity reached 95.28%, the specificity reached 95.33%, the accuracy reached 95.44%, and the F1 fraction was 95.36%, all of which were better than the improved model. Similarly, there is a slight increase in both parameter count and computational complexity; however, given the corresponding improvement in accuracy, this increase is warranted. The experimental results show that the improved model improves the ability to distinguish and recognize MCI and NC categories, reduces the probability of misidentifying MCI as normal aging, and enhances the ability to distinguish AD in the early stage.

**Figure 13:**
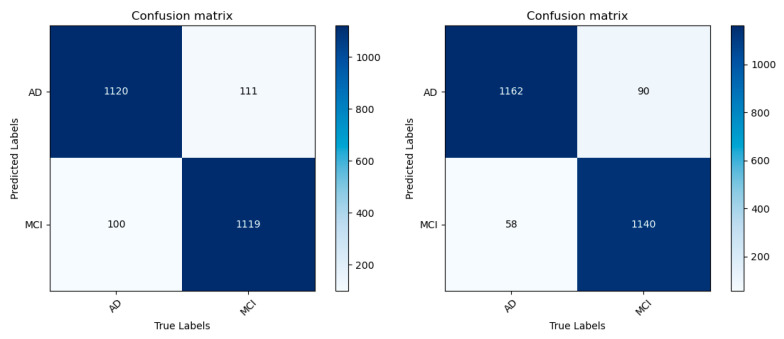
AD/MCI confusion matrices before and after model improvement.

**Figure 14:**
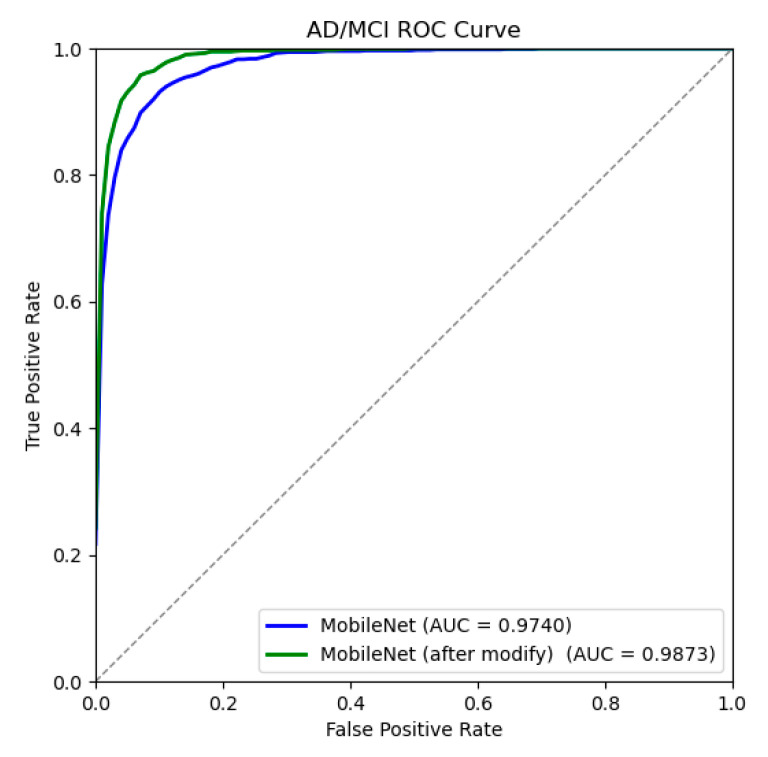
AD/MCI ROC curve before and after model improvement.

Similarly, a confusion matrix and ROC curve were drawn in the AD/MCI binary classification experiment, as shown in Figure 13 and Figure 14. Table 6 shows the results of each classification evaluation index. The results show that the accuracy of the improved model for AD/MCI classification reaches 93.96%, which is about 2.6 percentage points higher than before the improvement. Similarly, there is a slight increase in both parameter count and computational complexity; however, given the corresponding improvement in accuracy, this increase is warranted. The improved model can more accurately realize the AD/MCI classification task and can better distinguish the feature differences between the brain MR images of AD and MCI patients, providing an experimental basis for the early and accurate diagnosis of AD.

The three binary classification experiments validated that the model effectively differentiates between normal controls (NCs), mild cognitive impairment (MCI), and Alzheimer’s disease (AD) patients, demonstrating its capacity to discriminate across different stages of the disease. This outcome highlights the model’s generalizability across varied task scenarios, which is crucial for Alzheimer’s detection. Clinical applications rely on precise AD, MCI, and NC classification to improve diagnostic accuracy and optimize treatment timing. Furthermore, this foundation supports the extension to multi-class classification among AD, MCI, and NC groups.

### 4.4. Tripartite Experiments

In the process of AD diagnosis, the experiment of AD/MCI/NC triad classification expands the wide application capability of the AD automatic aided diagnosis algorithm model. In this section, the AD/MCI/NC ternary classification experiment is carried out, and the classification task is carried out based on the improved MobileNetV3 network model. The final model reaches a good classification level.

**Figure 15:**
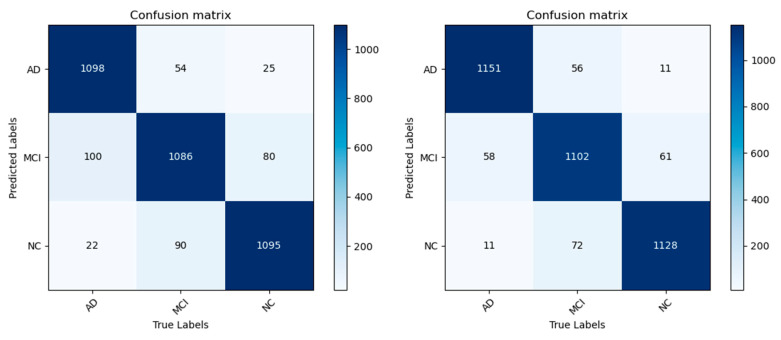
AD/MCI/NC confusion matrices before and after model improvement.

**Figure 16:**
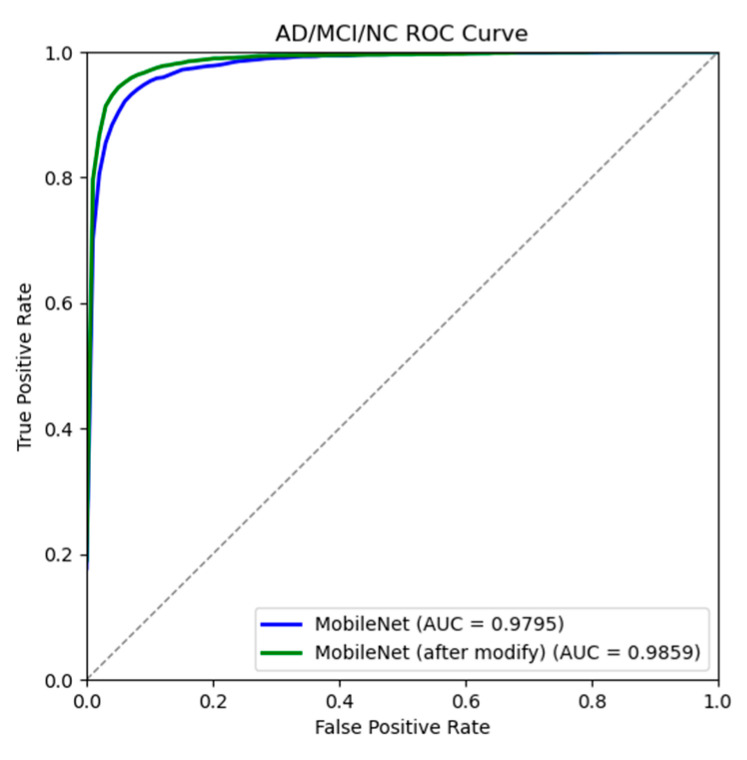
AD/MCI/NC ROC curve before and after model improvement.

Table 7 and Table 8 show the results of different classification evaluation indicators before and after model improvement. Comparing the two tables, it is found that all indicators of the improved model have been greatly improved, indicating that the improved model can more accurately identify the disease characteristics of different stages of AD and can more accurately distinguish the disease differences between different AD states. Figure 15 and Figure 16 plot the relevant confusion matrix and ROC curve. The results show that the improved model has better performance in three categories and can assist the automatic diagnosis of AD.

Both binary and term-based classification experiments were segmented based on the improved 3D DenseNet model and then classified based on the improved MobileNetV3 model. The experimental results showed excellent classification performance, which can effectively realize the accurate diagnosis of AD. MobileNetV3 is a lightweight CNN model, which can not only reduce the amount of computation and relieve the pressure on computer computing power but also ensure high classification accuracy, improve the overall efficiency of the experiment, and provide a reasonable and effective scheme for realizing accurate and convenient AD automatic assisted diagnosis. Thus, it can better assist doctors to make more suitable intervention and treatment measures for patients.

### 4.5. Experimental Comparison and Analysis

In order to verify the good performance of the lightweight MobileNetV3 model used for AD automatic classification and its improvement, this paper first selected two mainstream lightweight CNN models (EfficientNetB0 and ShuffleNetV2) for relevant experiments. Table 9 shows the experimental results obtained after four lightweight CNN models were trained and tested based on AD classification datasets in various classification experiments. In the longitudinal comparison, the improved MobileNetV3 model proposed in this paper has the best performance in the classification task of AD among the lightweight CNN models.

Then, it was verified that the image enhancement of AD classification data based on DAGAN can effectively improve the classification effect of the model. Comparison experiments are conducted on whether the model adopts the enhanced data and keeps other network structures unchanged to verify its effectiveness. The experimental results are listed in Table 10. The experimental results show that the image enhancement of AD image data is conducive to improving the feature extraction ability of the MobileNetV3 model. Thus, AD can be more effectively assisted in automatic diagnosis.

Then, it was verified that replacing the CA attention mechanism of SE can effectively improve the model performance. Only the SE attention module in the model is replaced, respectively, and other network structures are kept unchanged to verify the effect, as shown in Table 11. According to the results in the table, MobileNetV3 uses CA attention to replace the original SE attention module, which greatly improves the accuracy of various classification experiments.

### 4.6. Ablation Experiment

In order to verify that the improved algorithm model can significantly improve the classification accuracy and performance of AD, MCI, and NC, an ablation experiment was conducted on the model before and after the improvement. Table 12 lists the experimental results. It can be observed from the table that the overall performance of the model can be improved to varying degrees by replacing the CA attention module with the SE attention module, using hollow convolution to expand the convolution kernel receptive field, improving the fully connected layer to simplify the calculation, and preventing overfitting of the model. Among them, the combination of CA attention and void convolution DC has a relatively large improvement on the model, reflecting the significant advantages that the attention mechanism can capture more critical disease feature information and void convolution can acquire deeper features by expanding the receptive field. Through the combination of these optimization methods, the improved MobileNetV3 model can significantly improve the recognition ability of AD classification data so that it can assist doctors to realize AD diagnosis more accurately and effectively and also provide deep learning application schemes and technical support for the realization of portable AD automatic auxiliary diagnosis services.

## 5. Discussion

In this study, we proposed an Alzheimer’s disease detection method based on 3D DenseNet for segmentation and MobileNet for classification. While this approach has demonstrated certain advantages in model efficiency and accuracy, it still faces limitations in terms of generalization. Medical imaging datasets are typically small and exhibit uneven data distribution, which may cause the model’s performance to be unstable across different datasets, thereby limiting its applicability in real-world clinical settings.

In future research, multimodal data fusion could be an effective approach to enhance the model’s robustness and diagnostic accuracy. Alzheimer’s disease diagnosis often requires a combination of information sources, such as MRI, PET imaging data, genetic information, and cognitive assessments. Integrating these multimodal inputs can provide the model with richer contextual information, improving its ability to capture disease characteristics and identify early-stage abnormalities. This multimodal fusion approach not only holds promise for improving model generalizability but also offers more comprehensive diagnostic support for clinical applications. Additionally, Alzheimer’s disease is a progressive condition, and integrating longitudinal data over time into the model may improve early detection capabilities. Studies have shown that incorporating structural and functional changes across multiple time points can enable a more accurate diagnosis and prediction of early-stage symptoms. Developing models capable of analyzing time-series data could thus provide more precise and timely predictions for Alzheimer’s disease.

## 6. Conclusions

In this paper, MRI technology and deep learning methods were applied to the automatic assistant diagnosis of AD based on brain MR images, and a two-stage research method was proposed. In the diagnosis of AD, WM, GM, and CSF of the acquired brain MR images need to be segmented first so as to facilitate the model training to quickly and accurately locate and find the characteristics of the disease. Therefore, this paper proposes a method to automatically segment the classified data based on the improved 3D DenseNet model and then input the segmented data into the improved MobileNetV3 classification model for automatic classification to realize the automatic auxiliary diagnosis of AD. Through a comparison experiment and ablation experiment, the results show that the improved MobileNetV3 experimental model not only reduces the number of training parameters, improving the training speed, but also achieves a higher classification effect and better auxiliary diagnosis performance. However, this study only uses brain MRI single-modal data for AD classification, and multi-source data such as PET and MRI can also be obtained in subsequent studies. Multi-modal image data can be combined with multiple image feature information to extract key features of disease images more accurately, thus further improving the accuracy and performance of AD automatic auxiliary diagnosis. In this study, the 3D neural network classification model can be deeply explored, and the features of disease images can be more accurately located and captured by using the 3D spatial feature information of brain MR images. Future work should also explore the end-to-end AD automatic assisted diagnosis research, which can segment brain images and classify brain diseases in real time.

## Figures and Tables

**Table 1:** Classification datasets division.

	AD	MCI	NC	Total (100%)
Training set (64%)	3904	3936	3840	11,680
Validation set (16%)	976	984	960	2920
Testing set (20%)	1220	1230	1200	3650
Total (100%)	6100	6150	6000	18,250

**Table 2:** Dice ratio of improved 3D DenseNet for brain MR image segmentation.

Dice Ratio	Sub.1	Sub.2	Sub.3	Sub.4	Sub.5	Sub.6	Sub.7	Sub.8	Sub.9	Sub.10	Mean
GM	0.9309	0.8997	0.9409	0.9425	0.9145	0.9446	0.9313	0.9349	0.9276	0.9378	0.9305
WM	0.9768	0.9571	0.9806	0.9824	0.9835	0.9847	0.9766	0.9832	0.9730	0.9804	0.9778
CSF	0.8981	0.8579	0.9049	0.9148	0.9079	0.9151	0.8874	0.9083	0.8572	0.8992	0.8951

**Table 3:** Average Dice ratio of brain MR image segmentation based on various methods.

Methods	CSF	WM	GM	Mean
FSL	0.6480	0.9149	0.8133	0.7921
MIPAV	0.6430	0.9066	0.8255	0.7917
SPM	0.6711	0.8278	0.7832	0.7607
RF	0.8017	0.9467	0.8981	0.8822
U-Net	0.8772	0.9501	0.9079	0.9117
3D DenseNet	0.8721	0.9524	0.9126	0.9124
Improved 3D DenseNet	0.8951	0.9778	0.9305	0.9345

**Table 4:** AD/NC experiment results of model before and after improvement.

Methods	Accuracy	Sensitivity	Specificity	Precision	F1 Score	Parameters	MFlops
MobileNetV3	94.75%	96.56%	92.92%	93.27%	94.89%	2.9M	66
Improved MobileNetV3	97.85%	97.62%	98.08%	98.11%	97.86%	3.1M	80

**Table 5:** MCI/NC experiment results of model before and after improvement.

Methods	Accuracy	Sensitivity	Specificity	Precision	F1 Score	Parameters	MFlops
MobileNetV3	92.51%	91.38%	93.67%	93.67%	92.51%	2.9M	65
Improved MobileNetV3	95.31%	95.28%	95.33%	95.44%	95.36%	3.1M	79

**Table 6:** AD/MCI experiment results of model before and after improvement.

Methods	Accuracy	Sensitivity	Specificity	Precision	F1 Score	Parameters	MFlops
MobileNetV3	91.39%	91.80%	90.98%	90.98%	91.39%	2.9M	67
ImprovedMobileNetV3	93.96%	95.25%	92.68%	92.81%	94.01%	3.1M	81

**Table 7:** AD/MCI/NC experiment results of MobileNetV3 model.

	Sensitivity	Specificity	Precision	F1 Score
AD	90.00%	96.75%	93.29%	91.62%
MCI	88.29%	92.56%	85.78%	87.02%
NC	91.25%	95.43%	90.72%	90.98%

**Table 8:** AD/MCI/NC experiment results of improved MobileNetV3 model.

	Sensitivity	Specificity	Precision	F1 Score
AD	94.34%	97.24%	94.50%	94.42%
MCI	89.59%	95.08%	90.25%	89.92%
NC	94.00%	96.61%	93.15%	93.57%

**Table 9:** Experimental results of different lightweight CNN models.

Methods	AD/NC	MCI/NC	AD/MCI	AD/MCI/NC	Model Size
EfficientNetB0	94.73%	91.41%	90.34%	89.39%	4.21 MB
ShuffleNetV2	95.72%	92.73%	91.82%	90.47%	5.14 MB
MobileNetV3	94.75%	92.51%	91.39%	89.84%	5.84 MB
Improved MobileNetV3	97.85%	95.31%	93.96%	92.63%	5.28 MB

**Table 10:** Experiment results of data enhancement.

	AD/NC	MCI/NC	AD/MCI	AD/MCI/NC
MobileNetV3	93.62%	91.43%	90.41%	88.89%
MobileNetV3 + DAGAN	94.75%	92.51%	91.43%	89.88%

**Table 11:** Experiment results of different attention mechanisms.

Methods	AD/NC	MCI/NC	AD/MCI	AD/MCI/NC
MobileNetV3 + SE	96.25%	93.94%	92.67%	91.10%
MobileNetV3 + CBAM	97.28%	94.89%	93.52%	91.98%
MobileNetV3 + CA	97.85%	95.31%	93.96%	92.63%

**Table 12:** Experiment results of ablation.

Methods	AD/NC	MCI/NC	AD/MCI	AD/MCI/NC
MobileNetV3	94.75%	92.51%	91.39%	89.84%
MobileNetV3 + CA	95.90%	93.64%	92.36%	90.79%
MobileNetV3 + DC	95.68%	93.36%	92.20%	90.61%
MobileNetV3 + Improved FC	95.28%	92.92%	91.77%	90.19%
MobileNetV3 + CA + DC	96.82%	94.15%	92.95%	91.35%
MobileNetV3 + CA + Improved FC	96.21%	93.83%	92.60%	91.02%
MobileNetV3 + DC + Improved FC	95.97%	93.68%	92.51%	90.92%
MobileNetV3 + CA + DC + Improved FC	97.85%	95.31%	93.96%	92.63%

## Data Availability

Data are available at: https://adni.loni.usc.edu/, accessed on 15 January 2023.
